# Lung Function in Users of a Smoke-Free Electronic Device With HeatSticks (iQOS) Versus Smokers of Conventional Cigarettes: Protocol for a Longitudinal Cohort Observational Study

**DOI:** 10.2196/10006

**Published:** 2018-11-05

**Authors:** Almaz Sharman, Baurzhan Zhussupov, Dana Sharman, Irina Kim, Elmira Yerenchina

**Affiliations:** 1 Academy of Preventive Medicine of Kazakhstan Clinical Research Unit Almaty Kazakhstan; 2 Synergy Research Group Kazakhstan Almaty Kazakhstan

**Keywords:** COPD, iQOS with HeatSticks, exacerbations, respiratory symptoms, CT scan, COPD assessment test

## Abstract

**Background:**

Chronic obstructive pulmonary disease (COPD) is a global public health problem. It is the third-leading cause of death in the world, the fourth leading cause of death in Kazakhstan, and is strongly associated with smoking. Smoking cessation reduces the severity of respiratory symptoms and COPD exacerbations. Heated tobacco products, such as HeatSticks heated by the iQOS device, a smoke-free electronic device, may serve as less risky alternatives to conventional combustible cigarettes.

**Objective:**

The purpose of this study is to evaluate frequency of exacerbations, respiratory symptoms, physical exercise intolerance, and abnormal lung functions, as well as other parameters and comorbidities among men and women aged 40-59 residing in Almaty, Kazakhstan, who use iQOS with HeatSticks compared to smokers of conventional cigarettes.

**Methods:**

This is a 5-year single-center cohort observational study. It includes two cohorts of participants consisting of men and women aged 40-59 residing in the city of Almaty, Kazakhstan: (1) smokers of combustible cigarettes (control group) and (2) users of iQOS with HeatSticks (exposure group). The study has baseline and periodic (ie, annual) comprehensive clinical assessments, as well as continuous COPD case-finding activities and registration of acute respiratory exacerbations over the course of the 5-year observation period. Study measures include spirometry, chest computed tomography, electrocardiography, physical exams, laboratory testing of serum for biomarkers of inflammation and metabolic syndrome, anthropometry, and the 6-minute walk test. Information about COPD symptoms will be collected using the COPD Assessment Test.

**Results:**

Participant recruitment began December 2017, and enrollment is expected to last until late summer 2018.

**Conclusions:**

This is the first cohort observational study in Kazakhstan to assess differences in lung function between users of the heated tobacco product, iQOS with HeatSticks, and smokers of conventional combustible cigarettes. The study results will add to knowledge on whether switching from combustible cigarettes to iQOS with HeatSticks affects respiratory symptoms and diseases, including the development and progression of COPD.

**Trial Registration:**

ClinicalTrials.gov NCT03383601; https://clinicaltrials.gov/ct2/show/NCT03383601 (Archived by WebCite at http://www.webcitation.org/72BYoAKxa)

**International Registered Report Identifier (IRRID):**

PRR1-10.2196/10006

## Introduction

Chronic obstructive pulmonary disease (COPD), the fourth leading cause of death in Kazakhstan, is a global public health problem. It is the third leading cause of death globally, accounting for 3.2 million deaths worldwide in 2015 [1]. In Kazakhstan, an expected 1.4 million people may have COPD based on estimations from neighboring countries [2]. COPD negatively affects the quality of life and is a major health care burden [3]. It is the third leading cause of hospital readmission within 30 days [4]. Cigarette smoke is the most common risk factor for COPD [5]. COPD is traditionally defined by airflow obstruction and includes emphysema, gas trapping, and chronic bronchitis [6]. Systemic effects (eg, on heart and muscles) and associated comorbidities (eg, heart failure, metabolic disorders, sleep apnea syndrome, and depression) may complicate the course of disease, posing challenges in the management of COPD [7-9].

Recently, we conducted a cross-sectional study of COPD among men and women aged 40-59 who currently smoke cigarettes, do not smoke, and stopped smoking 1-5 years ago [10]. We demonstrated that based on the COPD Assessment Test (CAT), respiratory symptoms are common in current smokers who have spirometric values that are generally considered to be within the normal range. We identified a relatively low percentage of participants with obstructed respiratory functions on spirometry. It was higher among current smokers (5.5%) compared to former and nonsmokers (3% among both groups). We found that 42% of current smokers had COPD symptoms based on a CAT score of ≥10—a prevalence of symptoms that was far greater than that among former smokers and controls who never smoked (17% and 12.5%, respectively). In addition, smoking cessation significantly reduced functional exercise incapacity, that is, inability to walk 450 meters within 6 minutes in the 6-Minute Walk Test (6MWT; 11% among ex-smokers compared to 16.7 % among current smokers).

Our findings agree with and extend previously published data, including studies that document exacerbation-like events in smokers without airway obstruction [11,12]. Many current or former smokers, despite normal spirometry values, have clinical symptoms and findings that are consistent with a chronic lower respiratory disease similar to COPD. In addition, these symptomatic current or former smokers with preserved pulmonary function had a higher risk of respiratory exacerbations or abnormalities on a chest computed tomography (CT) scan or shorter 6-minute walk distances than asymptomatic current or former smokers with preserved pulmonary function. The CAT is a clinically useful tool that can identify smokers at risk for exacerbations [11].

Smoking cessation reduces the severity of respiratory symptoms and slows the mean rate of lung function decline but does not eliminate the risk of progressive lung disease [13]. In our cross-sectional study, we demonstrated negative association between smoking cessation and activity limitations and positive association between smoking of combustible cigarettes and evidence of airway disease. As compared to never-smokers, current and former smokers had elevations in all components of the CAT score: cough, phlegm, chest tightness, breathlessness going up hills/stairs, activity limitation at home, confidence leaving home, sleep, and energy. At the same time, those parameters were lower among those who stopped smoking 1-5 years ago compared to those who continued smoking.

Alternative tobacco products to conventional cigarettes have come on the global market with claims of being “modified risk” tobacco products. These electronic devices heat the tobacco instead of burning it to supposedly deliver fewer toxins than cigarette smoke. These heated tobacco products include HeatSticks, a specially designed heated tobacco unit that contains tobacco heated up to 350˚C by the iQOS device. The product was developed by Philip Morris International, Inc, which claims that the vapor from HeatSticks heated by the iQOS device contains 90%-95% less harmful and potentially harmful compounds and is 90%-95% less toxic than the smoke of a reference combustible cigarette. Results of a 3-month reduced-exposure study in Japan showed that a reduction in 15 biomarkers of exposure to 15 harmful and potentially harmful compounds for the smokers who switched to a heated tobacco product for the duration of the study approaches the reduction in the same biomarkers for smokers who quit for the duration of the study [14]. Polosa et al showed that electronic cigarettes (ie, a battery-operated device that emits doses of vaporized nicotine) might improve COPD outcomes, including subjective respiratory outcomes as well as annual exacerbation rate [15]. We hypothesize that participants who use iQOS with HeatSticks will have less prevalent presence of respiratory symptoms, have better functional exercise capacity, and experience fewer exacerbations compared to those who smoke combustible cigarettes.

## Methods

### Study Design

The goal of this study is to evaluate whether the presence of respiratory symptoms, functional exercise incapacity, and COPD exacerbation rate across time are the same between the exposure group (users of iQOS with HeatSticks) and the control group (smokers of combustible cigarettes) through hypothesis testing.

In addition to this confirmatory goal, we would like to explore trends in spirometry results, chest CT scan results, prevalence of impaired quality of life, metabolic syndrome, abnormalities found by electrocardiography (ECG), physical exams, and serum lab tests among those who use iQOS with HeatSticks and those who currently smoke combustible cigarettes.

Exacerbation history will be collected prospectively (every 3 months) with the use of the adapted version of our proprietary SymptoMaster (Web, iOS, and Android) [16], and Medintel (Web) apps [17]. Symptoms history will be analyzed by clinical investigators. Should there be symptoms identified that are relevant to COPD, clinical investigators will approach health care organizations (serving participants in primary health care catchment area) to obtain ambulatory records of the use of antibiotics and/or systemic glucocorticoids or a health care utilization event.

Our primary confirmatory outcome variables are (1) presence of respiratory symptoms defined by CAT≥10, (2) functional exercise incapacity, and (3) exacerbation rate. The main exposure variable is smoking status (users of combustible cigarettes and users of iQOS with HeatSticks). The study groups are defined as:

1. Individuals (men and women) aged 40-59 years (inclusive) with a minimum of 10 pack-year smoking history who switched to and predominantly use the heated tobacco product iQOS with HeatSticks (exposure group). Predominant use of iQOS with HeatSticks is defined as >70% use.
2. Individuals (men and women) aged 40-59 years (inclusive) who are currently smoking combustible cigarettes with a minimum of 10 pack-year smoking history (control group)

Pack-years will be calculated by taking the average number of cigarettes smoked per day divided by 20 and multiplied by the number of years smoked.

The Global Initiative for Chronic Obstructive Lung Disease (GOLD) definition describes COPD exacerbations as an acute worsening of respiratory symptoms that results in additional therapy [18]. They are classified as mild (ie, treated with short-acting bronchodilators only), moderate (ie, treated with short-acting bronchodilators and/or oral corticosteroids), or severe (ie, patient requires hospitalization or emergency room visit). Severe exacerbations may also be associated with acute respiratory failure. Once signs of exacerbations are identified, special efforts will be made by the Kazakhstan Academy оf Preventive Medicine (KAPM) clinical investigators, coordinators, and the principal investigator to establish their significance (ie, mild, moderate, or severe), and relevance to COPD.

### Inclusion and Exclusion Criteria

The study’s inclusion criteria comprise the following: male or female, aged 40-59 years, smoking history ≥10 pack-years (for all groups), and ability to follow study procedures. The exclusion criteria consist of the following:

- pregnant women
- legally incapable individuals
- patients with history of chronic infectious and noninfectious lung disease except asthma (eg, pulmonary fibrosis, bronchiectasis, cystic fibrosis, tuberculosis) diagnosed prior to or during the first visit to the KAPM COPD Center
- previous surgical excision of at least one lung lobe (or having undergone a lung volume reduction procedure)
- active cancer of any localization under treatment
- suspected cancer of any localization
- metallic objects in the chest
- recent eye surgery (within 6 months prior to the visit)
- episode(s) of myocardial infarction within 6 months prior to the visit or another form of acute or chronic coronary heart disease, history of heart rhythm abnormality with episode of arrhythmia within 6 months prior to the visit or long lasting that requires continuous drug therapy
- acute episode of cerebrovascular ischemic attack within 12 months prior to the visit
- chest or abdominal surgery performed within 6 months prior to the visit
- contraindications to salbutamol or refusal to inhale salbutamol
- chest radiation therapy within 12 months prior to the visit
- radiology diagnostic procedures of chest within 6 months prior to the visit
- recent (6 weeks before visit) respiratory tract infection (colds, flus), fever of any etiology with increasing temperature >37^°^C at the time of the visit and 2 weeks prior to the visit
- significant history of alcohol abuse or consumption of more than recommended units of alcohol per week (28 units male and 21 units female)
- positive screening test for HIV antibodies or positive screening for TB, if available at the time of first visit
- elevated blood pressure (systolic) ≥160 mmHg at the time of visit
- employees of Philip Morris International and first-degree relatives who are employees of Philip Morris International

### Sample Size Calculation

Based on the results of the cross-sectional study, we demonstrated that smoking cessation alleviates activity limitations and airway disease caused by smoking of combustible cigarettes. This is evidenced by the prevalence of a CAT score of ≥10 among those who stopped smoking during the past 1-5 years at 17% compared to 42.8% among current smokers, and reduced inability to walk 450 meters within 6 minutes on 6MWT (11% compared to 16.7% among current smokers).

We hypothesize that switching from combustible cigarettes to iQOS with HeatSticks may reduce the risk of respiratory exacerbation and other COPD manifestations, somewhat similar to the effects of smoking cessation observed in the cross-sectional study.

In the cross-sectional study, duration of smoking abstinence in the second (ex-smoker) study group varied from 1-5 years. This is comparable with the length of the cohort study (5 years) that we propose. Therefore, we can use estimates of the outcome variables derived from the cross-sectional study to calculate required sample sizes that can identify statistically significant differences.

We suggest implementing a matched-pair cohort study design where a pair contains one iQOS with HeatSticks user and two conventional cigarette users matched by gender, age, education, and baseline exposure level (number of pack-year). By selecting this study design, we take into account the limited number of iQOS users from which the first (exposure) cohort is planned to be recruited.

To perform sample size calculation, we will assume that the associations we observed in the cross-sectional study between primary confirmatory outcomes and the smoking status (ex-smokers vs current combustible cigarette smokers) will be the same for iQOS users as compared to combustible cigarette smokers at the final visit after 5 years:

1. At the final visit, the odds ratio of association between the exposure and CAT score of ≥10 will equal 0.27 and a CAT ≥10 prevalence will be 42.8% in the control group.
2. At the final visit, odds ratio of association between the exposure and inability to walk 450 meters on the 6MWT will equal 0.62 and a 6MWT <450 meters prevalence will be 16.7% in the control group.
3. At the final visit, rate ratio of association between the exposure and exacerbations will equal 0.5 and annual exacerbation rate will be 0.1 in the control group based on conservative estimations from Woodruff et al [10]. We also assume that these associations will increase steadily from 1 to the above-indicated values. Sample size calculations for three primary confirmatory outcomes have been performed by simulations and the frequentist method based on the power and level of significance of a test. For each primary confirmatory outcome, we have generated data from the distribution assumed for the combustible cigarette smokers and the distribution for the iQOS with HeatSticks smokers (based on our assumptions of how their changes in respiratory symptoms prevalence, proportions of participants who walk less than 450 meters, and annual exacerbation rate might be different if switched from combustible cigarettes to iQOS with HeatSticks). We have used R [19] for simulations (1000 simulations per each primary confirmatory outcome variable) and fitting a generalized linear mixed-effects model (GLMM) on simulated data (function “glmer” in the package “lme4”). For sample size calculation, we have used a power value of 80%, two-sided significance level (alpha) 1.7% that accounts for multiple hypotheses testing, and 1:2 ratio for sample sizes of the experimental and control cohorts.

Then, we have adjusted the sample size for anticipated proportion of dropouts (taking into consideration a 10% annual dropout rate and the fact that already collected data relating to any participant who stops the study early will be analyzed). Sample size calculations are presented in Table 1.

As a result, the sample size of 1041 respondents is enough to detect significant differences in three primary confirmatory outcomes with at least 80% power. However, in addition to dropouts, we need to consider that participants may switch from one tobacco product to another, quit smoking, use several tobacco products at the same time, and the fact that a nonrandomized observational study needs to be adjusted for confounding factors. Even if most of these events do not cause subjects’ withdrawal, they lead to a decreased number of participants for further statistical analysis. Therefore, we need more participants to solve this issue. We propose recruiting 400 participants in the exposure (iQOS) cohort and 800 participants in the control (combustible cigarettes) cohort. The following sample size should allow us to achieve the confirmatory goal of the study: (1) iQOS with HeatSticks smokers (exposure group, N=400) and (2) conventional cigarette smokers (control group, N=800; total sample size=1200).

### Study Procedures

#### Recruitment

We will select 800 current smokers and 400 users of iQOS with HeatStick based on the inclusion criteria. The recruitment strategy for smokers of combustible cigarettes and participants who use iQOS with HeatStick includes word-of-mouth communication to friends and spouses of individuals who participated in the cross-sectional study, advertisements, social media engagement (ie, Facebook, Instagram, WhatsApp chats), and outreach to community groups. In addition, we will recruit users of iQOS with HeatSticks at iQOS stores in Almaty, Kazakhstan.

To achieve comparability of comparison groups at baseline, the matched-pair cohort study design will be implemented, where a pair contains one iQOS with HeatSticks user and two conventional cigarette users. The following variables will be utilized for matching: gender, age (±3 years), education (as a proxy for socioeconomic status), and number of pack-year (±5 pack-years) as the baseline exposure level (number of pack-year).

Once potential participants who meet inclusion criteria are identified, they will undergo further medical assessment at the KAPM COPD Center to test for exclusion criteria and to start baseline comprehensive assessment.

The study will include (1) baseline comprehensive assessment at outset of the study during the enrollment period, which is expected to last for about 6 months (4^th^quarter of 2017 to 1^st^-3^rd^quarters of 2018); (2) annual comprehensive assessments at 2^nd^-4^th^quarters of 2018-2022; (3) periodic prospective case finding and exacerbation history assessment on quarterly basis (every 3 months), semiannual, and annual basis; and (4) continuous COPD case-finding activities based on patient self-assessment and professional clinical assessment during periodic visits to KAPM dedicated primary care unit.

**Table 1:** Sample size calculations.

Primary confirmatory variable	Estimated value of outcome variable in exposure group	Estimated value of outcome variable in control group	Sample size exposed/ nonexposed	Adjusting sample size for anticipated dropouts
Symptomatic smokers defined by CAT ≥10	17%	42.8%	23/46	31/62
6MWT <450 m	11%	16.7%	260/520	347/694
Annual exacerbation rate	0.05	0.1	255/510	340/680

Baseline comprehensive assessment at outset of the study (4^th^quarter of 2017) and annual assessments (4^th^quarters of 2018-2020) will include the following:

- COPD assessment test
- 6MWT
- Short form-12 (SF-12) Quality of Life questionnaire
- spirometry (forced expiratory volume in 1 second [FEV1]/ forced vital capacity [FVC] bronchodilation test
- ECG
- body mass index
- CT scan of the chest
- COPD-related conditions and comorbidities identified by using our proprietary mHealth technologies: SymptoMaster and Medintel
- Stanford 25 comprehensive clinical assessment to identify clinical signs of COPD and comorbidities [20,21]
- laboratory testing for complete blood count (CBC), blood cholesterol level, high-density lipoproteins (HDL), low-density lipoproteins (LDL), triglycerides, C-reactive protein, fibrinogen, glucose
- biomarker testing for sRAGE, ICAM1, CCL20, and probably other biomarkers (to be determined based on validity and feasibility of each test)

One important feature for an efficient support tool is the ability to detect any symptom that can lead to a potential new COPD case finding and exacerbation. We will administer the CAT to identify COPD symptoms and quantify them during a stable phase of disease (>6 weeks after any exacerbation). The CAT is a validated 8-question health-status instrument with scores ranging from 0-40, with higher scores indicating greater severity of symptoms. GOLD uses a CAT score ≥10 as a threshold for more severe symptoms in consideration of treatment regimens [7].

In the context of COPD, an exacerbation is defined as a worsening of a patient’s symptoms from their usual stable state. The symptoms can then be analyzed by physicians using SmartHealth algorithms to detect the potential risk of an exacerbation. We will obtain an exacerbation history prospectively (ie, every 3 months) with the use of a structured questionnaire. Exacerbations will be defined on the basis of the use of antibiotic agents, systemic glucocorticoids, or a combination of both or a health care utilization event (ie, office visit, hospital admission, or emergency department visit for a respiratory flare-up). Severe exacerbations will be defined as interactions that lead to hospitalization or an emergency department visit. Exacerbations will be managed by the participants’ primary care physicians in close communication with study participants, their families, and health care organizations in their service areas of Almaty.

The inclusion of secondary endpoints (eg, lab tests, ECG, and clinical assessment) allows for generating further hypotheses for different effects of switching to iQOS. Schedule of enrollment, data collection, and assessment are shown in Tables 2 and 3.

All study participants in both cohorts will receive smoking cessation advice according to clinical standards endorsed by Kazakhstan’s Health Ministry, offered throughout the study at each visit. The participants will also be offered formal professional assistance on smoking cessation. At the screening visit, in conjunction with signing the informed consent form, clinical investigators will give smoking cessation advice. It will be free of charge (paid by the sponsor) and is set out in the informed consent form. It will include psychological support to eliminate nicotine dependency, and participants will be referred to a specialist commercial medical organization in Almaty. We anticipate that some smokers and iQOS users will quit and that some smokers will switch to a reduced-risk product during the study. Quitting and switching will not be considered as reasons for early withdrawal and will be accounted for in sample size calculations.

#### Spirometry

Spirometry data will be collected using the combined spirometry system, BTL-08 SPIRO. All spirometry studies will be reviewed centrally to ensure quality control. Bronchodilator responsiveness will be considered positive if the subject had a ≥12% change in FEV1 or FVC above pre-bronchodilator measurements [22].

Each spirometer to be used in this study will be tested and continuously standardized with a 3.0-liter syringe. Each clinical coordinator will be certified after spirometry training. Quality assessments will be made on each study.

Smokers will be categorized for analysis using the GOLD staging system according to the results on spirometry, which will be performed before and after two inhalations of salbutamol, 0.1 μg per inhalation. Among the criteria needed to make a diagnosis of COPD are deficits in the rate at which one can forcefully exhale. Most experts consider a low ratio (<0.70) of the FEV1 to the FVC after bronchodilator use to be a key diagnostic criterion [7].

Once the diagnosis of COPD has been established, the GOLD nomenclature grades severity according to the degree to which the measured FEV1 is lower than the patient’s predicted value [7]:

- GOLD stage 1 (mild disease): FEV1 ≥80% of the predicted value
- GOLD stage 2 (moderate disease): FEV1 ≥50% and <80% of the predicted value
- GOLD stage 3 (severe disease): FEV1 ≥30% and <50% of the predicted value
- GOLD stage 4 (very severe disease): FEV1 <30% of the predicted value

#### Assessment Test for Chronic Obstructive Pulmonary Disease

The CAT is a validated, short (8-item) questionnaire to be completed by study participants. Despite the fact that CAT is designed for patients with COPD, it can be used to measure respiratory symptoms among all participants including those who have preserved pulmonary function [10]. The CAT has a scoring range of 0-40, with the cut-off point equaling 10.

**Table 2:** Schedule of participants enrollment and baseline assessment, 2017-2018.

Test	Screening^a^	Baseline visit^b^
Informed consent process	X	
Review inclusion/exclusion criteria to determine study eligibility, including smoking status and iQOS use	X	
Review medical history	X	
Provide study requirements handout (explain study/visit requirements)	X	
Pregnancy test	X	X
Stanford 25 comprehensive clinical assessment		X
Spirometry (FVC^c^, FEV^d^, FEV1^e^, FEV1/ FVC, FEF^f^ 25-75, MEF^g^), bronchodilation test using salbutamol		X
CAT^h^		X
6MWT^i^		X
Short form-12 Quality of Life questionnaire		X
ECG^j^		X
Body mass index		X
Smoking status and exposures		X
Comprehensive physical assessment Stanford 25		X
Previous COPD^k^ exacerbations based on use of inhaled steroids, antibiotics, inhaled bronchodilators, steroids		X
Previous COPD exacerbations based on health care visits (office visits, admissions to emergency department or hospital)		X
Previous COPD severe exacerbations based on emergency department admission or hospitalizations		X
Laboratory testing for blood cholesterol level, CBC^l^ (25 parameters); HDL^m^, LDL^n^, triglycerides, C-reactive protein, fibrinogen, glucose, glycosylated hemoglobin		X
Biomarker testing for sRAGE^o^, ICAM1^p^, and CCL20^q^		X
Lung computer tomography		X

^a^Months –3 to 3.

^b^Months 0 to 3.

^c^FVC: forced vital capacity.

^d^FEV: forced expiratory volume.

^e^FEV1: forced expiratory volume in 1 second.

^f^FEF: forced expiratory flow.

^g^MEF: maximum expiratory flow.

^h^CAT: COPD Assessment Test.

^i^6MWT: 6-Minute Walk Test.

^j^ECG: electrocardiogram.

^k^COPD: chronic obstructive pulmonary disease.

^l^CBC: complete blood count.

^m^HDL: high-density lipoproteins.

^n^LDL: low-density lipoproteins.

^o^sRAGE:advanced glycosylation end-product receptor.

^p^ICAM1: intercellular adhesion molecule 1.

^q^CCL20: macrophage inhibitory protein 3a.

**Table 3:** Schedule of participants visits for data collection and monitoring, years 1-5.

Test	Year 1			Year 2				Year 3				Year 4				Year 5				
	Q^a^2	Q3	Q4	Q1	Q2	Q3	Q4	Q1	Q2	Q3	Q4	Q1	Q2	Q3	Q4	Q1	Q2	Q3		Q4
Stanford 25 comprehensive clinical assessment				X				X				X				X				
Spirometry (FVC^b^, FEV^c^, FEV1^d^, FEV1/ FVC, FEF^e^ 25-75, MEF^f^), bronchodilation test using salbutamol				X				X				X				X				
CAT^g^				X				X				X				X				
6MWT^g^				X				X				X				X				
Short form-12 Quality of Life questionnaire				X				X				X				X				
ECG^g^				X				X				X				X				
Body mass index				X				X				X				X				
Smoking status, exposures				X				X				X				X				
Laboratory testing for blood cholesterol level, CBC^j^ (25 parameters); HDL^k^, LDL^l^, triglycerides, C-reactive protein, fibrinogen, glucose, glycosylated hemoglobin				X				X				X				X				
Biomarker testing for sRAGE^m^, ICAM1^n^, and CCL20^o^				X				X				X				X				
Lung computer tomography				X				X				X				X				
Continuous health status monitoring, including home visits, phone, email, telemedicine, and COPD^p^ Center visits as necessary	X	X	X	X	X	X	X	X	X	X	X	X	X	X	X	X	X		X	X
Monitoring COPD exacerbations based on use of inhaled steroids, antibiotics, inhaled bronchodilators, steroids, health care visits (office visits, emergency department, or hospital)	X	X	X	X	X	X	X	X	X	X	X	X	X	X	X	X	X		X	X
Monitoring adverse events and serious adverse events using SymptoMaster self-assessment that triggers notification	X	X	X	X	X	X	X	X	X	X	X	X	X	X	X	X	X	X		X

^a^Q: quarter.

^b^FVC: forced vital capacity.

^c^FEV: forced expiratory volume.

^d^FEV1: forced expiratory volume in 1 second.

^e^FEF: forced expiratory flow.

^f^MEF: maximum expiratory flow.

^g^CAT: COPD Assessment Test.

^h^6MWT: 6-Minute Walk Test.

^i^ECG: electrocardiogram.

^j^CBC: complete blood count.

^k^HDL: high-density lipoproteins.

^l^LDL: low-density lipoproteins.

^m^sRAGE: advanced glycosylation end-product receptor.

^n^ICAM1: intercellular adhesion molecule 1.

^o^CCL20: macrophage inhibitory protein 3a.

^p^COPD: chronic obstructive pulmonary disease.

#### Computed Tomography

All study subjects will undergo inspiratory 64-channel CT scans of the chest with the following standard parameters: 0.8 mm reconstructed slice thickness, 0.4 mm slice interval, matrix size 512 x 512, range -500 to 1500, 120 kV, 40 mAs.

A trained professional research assistant will evaluate the scan for technical completeness, compliance with protocol, adequacy of inspiration, and presence of motion artifact. The stability of CT measurements for CT scanner used in the study will be monitored by monthly scanning using a custom phantom designed for this study.

While conducting this cross-sectional study, we identified an optimal quantitative CT assessment protocol by using Airway Inspector based on the Slicer computer program (Harvard University) for lung densitometry [23]. In addition to qualitative assessment of 3D lung reconstruction, Slicer software allows for quantitative measurements of lung tissue density based on Hounsfield units. Such a tool provides the opportunity for automated measurement and quantitative comparisons between study groups based on an objective emphysema scoring system.

#### Electrocardiography

A standard 12-lead ECG taken by Fukuda Denshi FX-8222 Cardimax will be performed for each study subject by employing strictly standardized procedures. Research staff will be trained to properly place electrodes. At least four cardiac cycles will be taken from each of 12 leads. The machine runs at 50 mm/sec. The following ECG parameters will be evaluated by a trained clinical researcher: waves and complexes, presence and description of ECG abnormalities including pathologic q-waves, ST elevation, ST depression, T-wave inversion, hypertrophy, QRS axis deviation, block, and arrhythmia. ECGs will be visually inspected for technical errors and interpreted by a qualified cardiologist. The prevalence of specific ECG abnormalities as well as grouped abnormalities will be reported for each study group. We will measure and analyze associations between COPD and ECG abnormalities, crude and adjusted by sex, age, and smoking status.

#### Physical Exam

Clinical investigators were trained to conduct the pulmonary (ie, percussion and inspection) exam and technique for listening to second heart sounds. Two Stanford Medicine 25 modules [20,21] will be used as study materials in hands-on sessions. The prevalence of individual pathological findings will be presented for each study group.

#### Anthropometry

Anthropometric measurements will include height, weight, waist circumference, heart rate, blood pressure, and pulse oximetry.

#### Six-Minute Walk Test

The 6MWT is a simple and effective test that measures the distance that a patient can quickly walk on a flat, hard surface in a period of 6 minutes. A 100-ft hallway is needed, and no exercise equipment or advanced training for technicians is required [24].

#### Laboratory Data

Blood donated by the study participants will be processed at the KAPM COPD Center for shipment, analysis, and intermittent (at -20°C) and long-term (deep freeze at -80°C) storage at HealthCity Laboratory in accordance with biobanking standards. The HealthCity Laboratory will perform the following assays: CBC, blood cholesterol level, HDL, LDL, triglycerides; glucose, hemoglobin A1C; C-reactive protein; and fibrinogen.

In addition, blood will be stored in order to perform testing for sRAGE, ICAM1, CCL20, and other potential biomarkers at a later stage. Biomarker testing will be done using enzyme-linked immunosorbent assay (ELISA) and/or aptamers analysis. Testing for other biomarkers, such as CDH1, CDH13, SERPINA7, Interleukin 16 (plasma level), and genotyping will also be considered depending on availability of the tests and their value in COPD diagnosis. We are also exploring technical capabilities for “-omics” studies, additional biomarkers, and tobacco-specific nitrosamines assay in urine.

Previously reported findings, particularly when combined with other studies of individual biomarkers, suggest a panel of blood biomarkers including sRAGE (advanced glycosylation end-product receptor), the biomarker of increased emphysema percentage in the lungs independent of gender, age, airflow limitation, body mass index, and current smoking status. Decreased ICAM1 levels correlate with increased severity of emphysema on CT scans, independent of smoking status, FEV1, and other covariates CCL20 (macrophage inhibitory protein 3a), inversely and significantly associated with emphysema. Interleukin 16 (IL-16; a multifunctional cytokine that has been associated with autoimmune and allergic diseases) is positively associated with age and body mass index and negatively associated with current smoking and emphysema in the upper lobes. An integrated “-omics” analysis in a very large cohort identified an association between decreased IL-16 and emphysema and discovered a novel IL-16 local expression quantitative trait loci (cis-eQTL) [25]. Thus, IL-16 plasma levels and IL-16 genotyping may be useful in a personalized medicine approach for lung disease.

### Collecting Data Using SmartHealth Technologies

KAPM’s SmartHealth technologies (SymptoMaster and Medintel) will be used to capture early symptoms of COPD and signs of COPD exacerbations and comorbidities. Our proprietary expert technology called SymptoMaster [16] will help patients establish the probable causes of the symptoms of diseases without assistance from a health care professional. Using a computer, mobile phone, or tablet, a patient inputs their symptoms into the system, which produces the most likely preliminary diagnosis. After receiving the diagnosis, a patient can refer to Medintel [17], an online library that contains information about 1000 common diseases, their causes, symptoms, ways to prevent them, and treat them. These technologies allow a patient to make an informed decision on whether they should seek immediate medical assistance by calling an ambulance or consult a doctor on their next routine visit. SmartHealth technologies facilitate monitoring patients’ conditions by clinical investigators.

### Computer-Assisted Personal Interviewing

KAPM has developed an electronic data capture system in the form of its proprietary computer-assisted personal interviewing app. The app is available for tablet personal computers and will be modified to be implemented in the cohort study to collect, store, and transmit data related to a personal survey interview. The questionnaire will be designed to collect data on possible COPD risk factors including history of smoking, current smoking, level of smoking exposure (in pack-year), passive smoking, occupational and environmental hazards, including dusts, chemicals, and indoor fuel pollution. The questionnaire will contain covariates: age, gender, ethnicity, education, occupation, and self-reported morbidity. It will also include questions to address COPD exacerbations and to record the use of combustible cigarettes, iQOS with HeatSticks, electronic cigarettes, and other alternatives, as well as iQOS switch date.

### Statistical Analysis and Data Management Plan

Appropriate descriptive statistics will be used to summarize required study elements overall and by cohort (eg, proportion, mean, standard error, median, and interquartile ranges). Exploratory graphical analysis will be done preliminary to numerical analysis. Histograms, two-dimensional scatterplots of raw data, will provide information on the univariate and bivariate distributions of the variables focusing on distribution of variables, relation between the variables, whether it is linear or nonlinear, etc. In addition, preliminary graphs will screen raw data by highlighting obvious data errors. Spaghetti plots, scatterplots of dependent variable scores versus the time variable with a separate line for each person, will explore likely models, especially whether effects are linear or not.

Generalized estimating equations (GEE) and GLMM will be used to assess the effect of exposure on outcome variables. Both GEE and GLMM are used to account for within-person correlation of observations due to repeated measures. GEE uncovers the population average effect of a covariate, whereas GLMM estimates the individual specific effect. For binary confirmatory outcomes, CAT score ≥10, and inability to walk 450 meters on the 6MWT, the logit transformation will be utilized as the model link function. To model the annual exacerbation rate (count data), we will use the logarithm transformation and the Poisson or quasi-Poisson distribution depending on mean-variance relationship.

Presence of the statistically significant “time by group” interaction coefficient in GEE/GLMM will be our focus. It will demonstrate that the primary outcomes in two cohorts will have been changed significantly and provide statistical evidence for the harm reduction hypotheses.

To adjust for confounders, we will examine distributions of all covariates in the exposed and control group and will use Cohen effect sizes [26] to identify whether participants’ characteristics are different between groups at baseline. The impact of a potential confounder will be confirmed on whether the adjustment for the confounding variable changes the estimate of association.

We will apply a three‐stage reporting framework [27]. Model 1 will report the crude analysis (without any adjustments for confounders), Model 2 will report the semi‐adjusted analysis (including the following a priori defined covariates of gender, age, number of pack-year at base-line), and Model 3 will present the fully adjusted model (including all the covariates as in Model 2 plus those covariates that have discrepancies across two cohorts and change the estimate of association). The matching variables can be ignored in the model if we have exactly two controls for each participant in the iQOS cohort. Because of dropouts and other reasons, that will be impossible to achieve. There will be some confounder effect remaining, which means we have to include matching variables in the analysis. Model diagnostic plots will be generated to test model assumptions, for example, normality of deviance residuals.

We will also use two methods of propensity score analysis (PSA)—stratification on the propensity score and inverse probability weighting using the propensity score [28]—to adjust for confounders that may vary in study groups.

For missing data analysis, we will use missing at random assumptions. Missing values will be handled by multiple multivariate imputation in R. We will analyze five copies of the data, each with missing values imputed, in the GEE/GLMM multivariate analysis. The estimates of association will then be averaged according to Rubin’s rule [29] to produce a single mean estimate and adjusted standard errors.

To address dual usage, switching, and quitting, we will use several methods. First, we will implement the intention-to-treatment strategy, that is, we will adjust only for baseline confounders and evaluate the exposure effect as if all study participants remain under the exposures at the baseline until end of follow‐up. Second, we will evaluate the exposure effect censoring switchers and quitters from the analysis. Third, particularly for dual usage, we will model two exposures, iQOS and conventional cigarette usage, as weighted sums of doses calculated in pack-years. Weights will depend on the importance of the different periods, where the closest period will have the highest weight.

Analysis will be done using R [19]. The Bonferroni correction of the significance level will be applied to account for three confirmatory hypotheses being tested in the study, so an alpha <.017 (0.05/3) will be considered significant.

Sensitivity analysis will be performed to assess consistency of the effect estimate by testing variations in underlying assumptions. First, we will assess potential biases due to unmeasured confounders or how strong should be an unmeasured confounder to explain the magnitude of the effect estimate. Second, we will consider the use of more than one cohort definition to ensure that the effect estimate is robust to the assumptions behind these definitions. For example, we will use a different cut-off for predominant use of iQOS with HeatSticks (>50%, >60%, >70%, >80%, >90%, 100%) based on averaging iQOS percentage use over time beginning from baseline. Third, there are several alternative statistical analysis approaches to evaluate the effect estimate. We will explore whether the choice of statistical method (GEE/GLMM/PSA) will influence the effect estimate. Finally, we will search how redefining the threshold (ie, different cut-off points for binary outcomes, CAT, and 6MWT) changes the effect estimate.

All study data will be stored at the Information Technology Unit of КАPM. Data will be entered through a Web-accessible system. The questionnaire will be designed as an HTML Web form and will be placed on the server so that it can be loaded and displayed through the client browser at the time of connection. Data analysis will be done only in aggregate. All data will be used for research purposes only, and no participant will be identified when the data are analyzed, presented, or published. No individually identifiable information will be published. The Ethics Committee of KAPM approved this study on December 5, 2017.

## Results

Participant inclusion began December 2017 and recruitment is expected to last until late summer 2018. When completed, the study results will be published in peer-reviewed scientific journals.

## Discussion

### Principal Considerations

To the best of our knowledge, this is the first longitudinal cohort observational study to demonstrate whether trends in the response variables across time differ between users of iQOS with HeatSticks (exposure group) and smokers of combustible cigarettes (control group).

We hypothesize that iQOS with HeatSticks may serve as a less risky alternative to combustible cigarettes and to other traditional tobacco products in a clinical setting. Specifically, we hypothesize that participants using iQOS with HeatSticks will have less prevalent presence of respiratory symptoms, have better functional exercise capacity, and experience fewer exacerbations compared to those who smoke combustible cigarettes.

Assuming that smokers who switch to iQOS do reduce their exposure to harmful and potentially harmful smoke compounds, our study will determine whether switching to iQOS actually reduces the development and progression of COPD, based on CT scans and conventional spirometry tests.

### Limitations

This study is observational. Despite the fact that we have collected an extensive set of data on possible confounders, unmeasured confounding variables may exist. The study cannot produce definitive proof of a cause-effect relationship between the exposures and health outcomes, as with any observational medical research. Participants may leave the study for different reasons, which can compromise the validity of the study, particularly if the cohort dropout rates are different or the participants who remain in the study are different from those who drop out.

### Conclusion

This is the first cohort study in Kazakhstan to evaluate differences between smoking of combustible cigarettes compared to using the heated tobacco product, iQOS with HeatSticks, and the effect on respiratory symptoms, functional exercise capacity, and exacerbation rate of COPD. The study results will add to knowledge on whether switching from combustible cigarettes to iQOS with HeatSticks affects respiratory symptoms and diseases including the development and progression of COPD.

## Abbreviations

6MWT: Six-Minute Walk Test
CAT: COPD Assessment Test
CBC: complete blood count
CCL20: macrophage inhibitory protein 3a
COPD: chronic obstructive pulmonary disease
CT: computed tomography
ECG: electrocardiogram
FEF: forced expiratory flow
FEV: forced expiratory volume
FEV1: forced expiratory volume in 1 second
FVC: forced vital capacity
GEE: generalized estimating equation
GLMM: generalized linear mixed model
GOLD: Global Initiative for Chronic Obstructive Lung Disease
HDL: high-density lipoproteins
ICAM1: intercellular adhesion molecule 1
KAPM: Kazakhstan Academy оf Preventive Medicine
LDL: low-density lipoproteins
MEF: maximum expiratory flow
PSA: propensity score analysis
SF-12: Short form-12
sRAGE: advanced glycosylation end-product receptor
